# Epi-Off versus Epi-On Corneal Collagen Cross-Linking in Keratoconus Patients: A Comparative Study through 2-Year Follow-Up

**DOI:** 10.1155/2018/4947983

**Published:** 2018-07-29

**Authors:** F. Cifariello, M. Minicucci, F. Di Renzo, D. Di Taranto, G. Coclite, S. Zaccaria, S. De Turris, C. Costagliola

**Affiliations:** ^1^Department of Medicine and Health Science, University of Molise, Campobasso, Italy; ^2^Casa di Cura “Villa Maria”, Campobasso, Italy; ^3^I.R.C.S.S. Neuromed, Pozzilli, Isernia, Italy

## Abstract

**Aim:**

To evaluate two different techniques of cross-linking: standard epithelium-off (CXL epi-off) versus transepithelial (CXL epi-on) cross-linking in patient with progressive keratoconus.

**Methods:**

Forty eyes from 32 patients with progressive keratoconus were prospectively enrolled from June 2014 to June 2015 in this nonblinded, randomized comparative study. Twenty eyes were treated by CXL epi-off and 20 by CLX epi-on, randomly assigned, and followed for 2 years. All patients underwent a complete ophthalmologic testing that included uncorrected and best corrected visual acuity, central and peripheral corneal thickness, corneal astigmatism, simulated maximum, minimum, and average keratometry, corneal confocal microscopy, Schirmer I and break-up time (BUT) tests, and the Ocular Surface Disease Index. Intra- and postoperative complications were recorded. The solution used for CXL epi-off comprised riboflavin 0.1% and dextran 20.0% (Ricrolin), whereas the solution for CXL epi-on (Ricrolin TE) comprised riboflavin 0.1%, dextran 15.0%, trometamol (Tris), and ethylenediaminetetraacetic acid. Ultraviolet-A treatment was performed with a UV-X system at 3 mW/cm^2^.

**Results:**

In both groups, a significant improvement in visual function (Group 1: baseline 0.36 ± 0.16 logMAR, two-year follow-up 0.22 ± 0.17 logMAR, *p*=0.01; Group 2: baseline 0.32 ± 0.18 logMAR, 2-year follow-up 0.27 ± 0.19 logMAR, *p*=0.01) was recorded. Keratometry remained unchanged in both groups. The mean corneal thickness showed a significant reduction (mean difference of corneal thickness: −55 micron and −71 micron, resp.). One-month after treatment, OSDI^©^ reached 13.56 ± 2.15 in Group 1 (*p*=0.03) and 11.26 ± 2.12 in Group 2 (*p*=0.04). At confocal microscopy, abnormal corneal nerve alterations were found in both groups. Fibrotic reaction (43.75%) and activated keratocyte (62.6%) were more commonly recorded in Group 1 than in Group 2 (25.0% and 18.75%), with *p*=0.668 and 0.356, respectively.

**Conclusion:**

Our findings demonstrate that both procedures are able to slow keratoconus progression. Both treatment modalities are equivalent in terms of results and related complications. CXL epi-on technique is preferable to CXL epi-off since it preserves the corneal thickness and improves visual acuity, also reducing the postoperative ocular discomfort during the study period.

## 1. Introduction

Corneal collagen cross-linking (CXL) has acquired nowadays popularity for the treatment of progressive corneal ectasia. This technique, stabilizing the progression of keratoconus, delays the need for keratoplasty and, thus, decreases the chance of corneal transplantation [1], through an increase of the corneal biomechanical strength [2]. The method was developed in 1997 at the Dresden University and was carried out in Italy for the first time in 2005 [3]. It involves the photoactivation of riboflavin with ultraviolet-A (UVA) radiation, that unfolds a series of photochemical reactions inducing inter- and intrafibrillary cross links in the corneal stromal lamellae [4]. In this way, the tensile strength of the cornea prevent further thinning and deformation of the corneal profile [5] and deterioration of vision and offers some degree of functional improvement [6]. The original protocol was an epithelium-off (epi-off) procedure: the central corneal epithelium (about 8 mm) is removed, and riboflavin solution (0.1% riboflavin-5-phosphate and 20% dextran T-500) is applied to the exposed corneal stroma. CXL epi-off has been modified over time in favor of a method that does not involve the epithelium debridement [7, 8], that is, the technique called epithelium-on CXL [9]. This new approach was introduced to reduce the postoperative side effects of conventional epi-off CXL, as corneal infections, subepithelial haze, sterile infiltrates, reactivation of herpetic keratitis, and endothelial damage [10]. Transepithelial technique combines some advantages of the conventional technique, maintaining a higher safety profile, but it increases the risk of failure with a possible need of further treatment [11]. In fact, the diffusion process of riboflavin in the stroma is limited by corneal epithelial tight junctions [12–14]. Riboflavin penetration through the epithelium can be increased by different strategies, such as changing the physicochemical properties of the riboflavin molecule by adding chemical enhancers in the riboflavin formulation [15] besides the mechanical disruption of corneal epithelium [16].

Iontophoresis is a novel noninvasive system aimed at enhancing the delivery of charged molecules into tissues using small electric current [17]. Riboflavin, in the formulation used for iontophoresis, is negatively charged [18]. This last technique seems to be the best option to lock the progression of keratoconus [19, 20]. Moreover, the UV penetration in this procedure is limited by the riboflavin impregnated intact corneal epithelium, making it safer compared to the epi-off.

The aim of this study has been to compare these two techniques and to evaluate the efficacy and safety of the two treatments.

## 2. Materials and Methods

Forty eyes from 32 patients with progressive keratoconus, followed at the University of Molise, Italy, from June 2014 to June 2015, were included in this nonblinded, randomized comparative study. The patients were randomly assigned to one of the two treatment groups (20 eyes were treated with CLX epi-off, and the other 20 eyes were treated with CLX epi-on). Progression of keratoconus was documented through a clinical and instrumental (topographic, pachymetric, or aberrometric) worsening in the previous 6 months of observation. Inclusion criteria were patients with evolving keratoconus, aged between 18 and 40 years, and with no evidence of corneal scarring. Exclusion criteria were patients with central and paracentral corneal opacities, Vogt's striae, previous intraocular surgery, history of herpetic keratitis, severe dry eye, and concomitant autoimmune diseases.

All patients underwent a complete ophthalmologic testing that included uncorrected and best corrected visual acuity (BCVA), central and peripheral corneal thickness, corneal astigmatism, simulated maximum, minimum, and average keratometry, corneal confocal microscopy, Schirmer I and break-up time (BUT) tests, and the Ocular Surface Disease Index. All intra and postoperative adverse events were recorded.

BCVA was determined using Snellen's chart and was converted to logarithm of the minimum angle of resolution (logMAR). Central and peripheral corneal thickness, *K* flat, *K* steep, and mean *K* were evaluated with Sirius (CSO spa, Firenze, Italy) and Pentacam® (OCULUS, Germany) topographs. Fibrotic reaction, corneal alteration of nerves, activated keratocytes, and corneal opacities were evaluated through confocal microscopy HRT III (Heidelberg Engineering, with Rostock Cornea Module, Heidelberg, Germany).

Cornea was examined for anterior thinning, the presence of inflammatory cells associated with the lenticule, and activation of corneal keratocytes, which may indicate the development of fibrosis [21, 22]. Images of corneal alteration nerves were acquired using the same illumination intensity and by focusing the microscope beneath the basal epithelium. Approximately five images were randomly selected for qualitative analysis from the basal epithelium. The subbasal nerve fibre was assessed. The confocal images were selected and analyzed by two clinicians (F.C. and M.M.). Activation of stromal keratocytes was assessed considering the degree of keratocyte activation by comparing the data obtained with a grading scale. The grading scale consisted of a series of images derived from concurrent studies of stromal keratocyte activation using confocal microscopy [23]. The examinations for corneal opacities were performed by two clinicans (F.C. and M.M.), and manual quantitative analysis of keratocytes was attempted twice: first by the examiner and then by an expert observer (C.C.). Two consecutive section images were taken at a depth of 150 *μ*m (measured from the epithelial surface) to subjectively estimate the pre- and postoperative anterior stromal cell density [24]. Lastly, eye discomfort was evaluated before treatment and one-month later in all subjects using the Ocular Surface Disease Index (OSDI) questionnaire. The unit of measurement was expressed in OSDI^©^ (Allergan, USA).

Epi-off CXL technique was performed after instilling 4% lidocaine for topical anesthesia and 1.0% pilocarpine to reduce the risk for ultraviolet light exposure. A 9.0 mm of corneal epithelium was mechanically removed. Riboflavin (0.1% in 20% dextran solution; Ricrolin; Sooft, Montegiorgio, Italy) was administered topically every 2 minutes for 30 minutes. The administration was continued every 2 minutes during UVA exposure. The cornea was exposed to UVA 370 nm light (UV-X System; Peschke Meditrade GmbH, Hünenberg, Switzerland) for 30 minutes at an irradiance of 3.0 mW/cm^2^. At the end of the procedure, ofloxacin and cyclopentolate eye drops were administered, and therapeutic contact lens (LAC ACUVUE-etafilcon A) was then applied and was removed 3 days after surgery. Topical tobramycin (four times daily for 1 week) and dexamethasone phosphate 0.1% (four times daily for 2 weeks) were prescribed. The therapeutic contact lens was removed three days later. Lubricating eye drops were prescribed for the following three months.

In the epi-on CXL group, corneal epithelial was not removed. Corneal imbibition was obtained with 0.1% riboflavin–15% dextran solution supplemented with Tris-hydroxymethylaminomethane and sodium ethylenediaminetetraacetic acid (Ricrolin TE; Sooft, Montegiorgio, Italy) applied every 5 minutes for 30 minutes. One drop of 1% pilocarpine was administered 30 minutes before treatment to reduce the risk for UVA exposure. Ten minutes later, a single dose of 4% lidocaine eye drops was administered to anaesthetize the cornea. Postoperatively, topical tobramycin (four times daily for 1 week) was prescribed. All patients were operated by the same surgeons (F.C. and M.M.). The patients were checked at day 1, 3, 7, and 15 and then after 1, 6, 12, and 24 months.

The study was approved by the Institutional Review Board (CTS, Department of Medicine and Health Sciences, University of Molise, Ref. no. 0001-05-2018; ClinicalTrials.gov Identifier: NCT01350323), and each patient gave their written informed consent after a detailed description of the procedure used and of the aim of the work.

## 3. Data Analysis

The significance between parameters was assessed by Student's *t*-test for paired values and chi-square test for nonparametric variables. The differences between the values of the two groups at the baseline and after therapy were evaluated with two sample *t*-test. Significance was set at *p* < 0.05.

## 4. Results

The mean age of patients in Group 1 was 24 ± 7 years (ranging from 15 to 31 years; 13 male/7 female). In Group 2, the mean age was 31 ± 10 years (ranging from 19 to 44 years; 16 male/4 female).

In Group I, BCVA at the baseline was 0.36 ± 0.16 logMAR and improved to 0.22 ± 0.17 logMAR in postoperative 2 years (*p*=0.01), whereas in Group 2, the values progressed from 0.32 ± 0.18 logMAR to 0.27 ± 0.19 logMAR (*p*=0.01). At the end of the follow-up, the difference between the two groups was also significant (*p*=0.01).

Mean *K* at the baseline was 46.19 ± 2.82 D and 47.00 ±2.79 D, respectively (Group 1 and Group 2); these two values in the postoperative period of 2 years remained unchanged: 46.16 ± 3.15 D (*p*=0.57) (Group 1) and 47.82 ± 4.06 D (Group 2) (*p*=0.10). In addition, the differences between the two values were not significant (*p*=0.08).

*K* steep and *K* flat at the baseline in Group 1 were, respectively, 47.75 ± 3.20 D and 44.62 ± 2.63 D and in Group 2 were 48.86 ± 3.27 D and 45.84 ± 2.53 D. Two years after treatment, *K* steep and *K* flat of Group 1 reached 47.76 ± 3.47 D (*p*=0.10) and 44.71 ± 3.03 D (*p*=0.33), whereas in Group 2, they were 49.75 ± 3.47 D (*p*=0.60) and 46.44 ± 3.67 D (*p*=0.25). On the contrary, at the end of the follow-up, the difference between the two groups was significant for both parameters (*p*=0.01).

Mean corneal thickness after 2 years significantly change in both groups (from 556.45 ± 23.56 *μ*m to 501.41 ± 21.91 *μ*m (*p*=0.01) and from 565.41 ± 31.91 *μ*m to 495.45 ± 43.16 *μ*m (*p*=0.01), resp.), but the difference between groups was not significant (*p*=0.10).

At the baseline, the OSDI^©^ score was 4.85 ± 1.18 and 4.98 ± 1.32, respectively (*p* not significant). After one month, the score increased to 13.56 ± 2.15 in Group 1 (*p*=0.01) and 11.26 ± 2.12 in Group 2 (*p*=0.04). The difference between the two groups was also significant (*p*=0.02).

Confocal microscopy data in both groups revealed corneal nerve alterations: 93.8% in the epi-off group (Group 1) and 87.5% in the epi-on group (Group 2). Activated keratocyte and fibrotic reaction in Group 1 represented 62.5% and 43.75%, respectively, whereas in Group 2, they were recorded in a significantly lowest percentage (25% and 18.75%; *p*=0.001 in both).

The main complications were observed in 3 patients: two in Group 1 (Vogt's striae in a patient; in another patient corneal haze type II) and one in Group 2 (Vogt's striae and in the same eye follicular conjunctivitis). Schirmer and BUT tests did not reveal lacrimation defects in both groups.

## 5. Discussion

Our findings report for the first time OSDI^©^ (Ocular Surface Disease Index) difference in patients who underwent CXL. Through the OSDI^©^, we evaluated the degree of ocular discomfort in the patient treated with the two different methods. The results show that the score was lower in patients of Group 2 (*p* < 0.05). At the baseline, the score was 4.85 ± 1.18 and 4.98 ± 1.32 OSDI^©^, respectively. After treatment, the score increased to 13.56 ± 2.15 OSDI^©^ in Group 1 and 11.26 ± 2.12 OSDI^©^ in Group 2. Despite the use of topical anesthetics, the greater mean postoperative pain in the epi-off CXL group compared to the epi-on CXL group probably depends on the exposure of the corneal nerves and the release of inflammatory mediators, especially prostaglandins and neuropeptides after epithelium removal and related healing processes [25] (Tables 1 and 2).

Statistical analyses show that the mean corneal thickness, two years later, change significantly in both groups (from 556.45 ± 23.56 *μ*m to 501.41 ± 21.91 *μ*m (*p*=0.01) and from 565.41 ± 31.91 *μ*m to 495.45 ± 43.16 *μ*m (*p*=0.01), resp.), as already reported [20, 26, 27]. Although epithelial remodeling and stroma edema disappear few days after treatment, it has been reported to be responsible of corneal thickness changes also over a longer time [28]. This could justify the rethickening to preoperative levels 12 months after surgery reported in the previous studies, especially in the epi-on procedure [9, 18, 25, 27]. On the contrary, our findings demonstrate that corneal thickness decreases two-year postoperatively. This suggests the involvement of different factors, that is, compression of collagen fibrils, changes in both corneal hydration and glycosaminoglycans synthesis, and keratinocyte apoptosis, that alone or in combination may play a detrimental role in the corneal rethickening [28].

A significant increase in BCVA compared to the baseline was recorded in both groups (*p*=0.01). However, the epi-on group exhibits a better improvement compared to the epi-off group at the end of the follow-up (*p*=001) (Table 3). Our results are consistent with those achieved by three previous randomized clinical trials [18, 27, 29], which demonstrated a more important recovery of BCVA in the epi-on group versus the epi-off. However, it has been shown that, for progressive keratoconus patients, the standard cross-linking procedure yields better results and increases the chances of stopping the disease's progression in the long term [30]. This discrepancy could be explained assuming that the effects of cross-linking mainly reflects the biomechanical impact on stiffening the thinning cornea rather than the reforming cornea shape [31]. Therefore, the significant difference in BCVA after two years between the two study groups is more easily understood (Tables 4 and 5).

The activated keratocyte and fibrotic reaction are more frequent in Group 1 patients. This might be due to a rearrangement of the corneal epithelium secondary to the treatment or more likely to the much deeper cross-linking activity in the epi-off group [14, 32].

Few side effects occurred in our study: in Group 1, two patients had complications (Vogt's striae and type II corneal hazes) and in Group 2, only one eye developed Vogt's striae at the apex of keratoconus. On the contrary, several complications have been reported in other previous series, especially after epi-off CXL, such as clinically significant corneal haze, endothelial damage, and sterile infiltrate infections [33, 34]. Lastly, the most significant complications after epi-off CXL are pain and photophobia, which required placement of bandage contact lens, sunglasses, and analgesia. Our study showed that these two important postoperative complications were minimal in the epi-on CXL patients, as assessed by the OSDI^©^ questionnaire.

The limit of this study is the small number of patients in each group; the strengths are the prospective design, the long term follow-up (two years), and the evaluation of OSDI^©^.

Despite the different penetration stroma demonstrated in other studies, the clinical outcomes after CLX epi-off and epi-on procedures show that keratoconus was relatively stable after 24 months, and no differences were observed comparing the two procedures. Moreover, our findings demonstrate that the CXL epi-on technique is preferable to CXL epi-off since it reduces postoperative ocular discomfort, maintaining the same profile of safety and efficacy.

## Figures and Tables

**Table 1:** Group 1 (epi-off): comparison of analyzed parameters (mean ± SD) before and after treatment at the end of the follow-up (2 years).

	Preoperative	Postoperative	*p* value
Mean corneal thickness (*µ*m)	556.45 ± 23.56	501.41 ± 21.91	0.01^*∗*^
*K* flat (D)	44.62 ± 2.63	44.71 ± 3.03	0.33
*K* steep (D)	47.75 ± 3.20	47.76 ± 3.47	0.10
Mean *K* (D)	46.19 ± 2.82	46.16 ± 3.15	0.57
BCVA (logMAR)	0.36 ± 0.14	0.22 ± 0.12	0.01^*∗*^
OSDI© (before and one-month later)	4.85 ± 1.18	13.56 ± 2.15	0.01^*∗*^

BCVA: best corrected visual acuity; OSDI: Ocular Surface Disease Index; ^*∗*^Student's *t*-test for paired values.

**Table 2:** Group 2 (epi-on): comparison of analyzed parameters (mean ± SD) before and after treatment at the end of the follow-up (2 years).

	Preoperative	Postoperative	*p* value
Mean corneal thickness (*µ*m)	565.41 ± 31.91	495.45 ± 43.16	0.01^*∗*^
*K* flat (D)	45.84 ± 2.53	46.44 ± 3.67	0.25
*K* steep (D)	48.86 ± 3.27	49.75 ± 3.47	0.60
Mean *K* (D)	47.00 ± 2.79	47.82 ± 4.06	0.10
BCVA (logMAR)	0.32 ± 0.16	0.27 ± 0.13	0.01^*∗*^
OSDI© (before and one-month later)	4.98 ± 1.32	11.26 ± 2.12	0.04^*∗*^

BCVA: best corrected visual acuity; OSDI: Ocular Surface Disease Index; ^*∗*^Student's *t*-test for paired values.

**Table 3:** Epi-off versus epi-on after 2-year follow-up (mean ± SD).

	Epi-off	Epi-on	*p* value
Mean corneal thickness (*µ*m)	501.41 ± 21.91	495.45 ± 43.16	0.10
*K* flat (D)	44.71 ± 3.03	46.44 ± 3.67	0.01^*∗*^
*K* steep (D)	47.76 ± 3.47	49.75 ± 3.47	0.01^*∗*^
Mean *K* (D)	46.16 ± 3.15	47.82 ± 4.06	0.08
BCVA (logMAR)	0.22 ± 0.12	0.27 ± 0.13	0.01^*∗*^
OSDI© (before and one-month later)	13.56 ± 2.15	11.26 ± 2.12	0.02

BCVA: best corrected visual acuity; OSDI: Ocular Surface Disease Index; ^*∗*^Student's *t*-test for paired values.

**Table 4:** Ocular Surface Disease Index (OSDI©).

	All of the time	Most of the time	Half of the time	Some of the time	None of the time
*Have you experienced any of the following during the last week:*					
(1) Eyes that are sensitive to light?	4	3	2	1	0
(2) Eyes that feel gritty?	4	3	2	1	0
(3) Painful or sore eyes?	4	3	2	1	0
(4) Blurred vision?	4	3	2	1	0
(5) Poor vision?	4	3	2	1	0
	Subtotal	Score			A

*Have problems with your eyes limited you in performing any of the following during the last week:*					
(6) Reading	4	3	2	1	0
(7) Driving at night?	4	3	2	1	0
(8) Working with a computer or bank machine (ATM)?	4	3	2	1	0
(9) Watching TV?	4	3	2	1	0
	Subtotal	Score			B

*Have your eyes felt uncomfortable in any of the following situations during the last week:*					
(10) Windy conditions?	4	3	2	1	0
(11) Places or areas with low humidity (very dry)?	4	3	2	1	0
(12) Areas that are air conditioned?	4	3	2	1	0
	Subtotal	Score			C

Add subtotals A, B, and C to obtain the result.

**Table 5:** Postoperative confocal microscopy in epi-off versus epi-on patients after 2-year follow-up.

	Epi-off (%)	Epi-on (%)	*p* value
Corneal nerve alteration	93.80	87.50	Not significant
Fibrotic reaction	43.75	25.00	<0.01^*∗*^
Alterated keratocyte	62.50	18.75	<0.001^*∗*^

^*∗*^Chi-square test for nonparametric variables.
